# Diagnostic performance of serum CA-125 and ultrasonography in ovarian tumour differentiation

**DOI:** 10.6026/973206300220764

**Published:** 2026-02-28

**Authors:** Halima Khanam, Mehriban Amatullah, Rebeka Sultana M.S.T, Saraban Tohura, Farzana Sharmin, Naznin Akter Zahan, Irin Haque, Romena Afroj

**Affiliations:** 1 Department of Gynaecological Oncology, Bangladesh Medical University, Dhaka, Bangladesh; 2 Department of Obstetrics and Gynecology, Bangladesh Medical University, Dhaka, Bangladesh; 3 Department of Obstetrics and Gynecology, Aalok Mother and Childcare Hospital, Dhaka, Bangladesh; 4 Gynaecological Oncology, Directorate General of Health Services (DGHS), Dhaka, Bangladesh; 5 Department of Obstetrics and Gynecology, Directorate General of Health Services (DGHS), Dhaka, Bangladesh

**Keywords:** Ovarian tumour, ultrasonography, CA-125, diagnostic accuracy

## Abstract

Early differentiation of benign versus malignant ovarian tumours is crucial for optimal management but remains challenging in many clinical settings. This analytical cross-sectional study at Rangpur Medical College Hospital, Bangladesh, included 62 surgically managed ovarian tumours (48 benign, 14 malignant) evaluated preoperatively by transabdominal ultrasonography and serum CA-125. Ultrasonography alone yielded a sensitivity of 57.42%, specificity 89.50%, PPV 61.84%, NPV 87.42% and accuracy 82.17%. Serum CA-125 showed better performance, with a sensitivity 71.42%, specificity 93.75%, PPV 76.92%, NPV 91.83% and accuracy 88.70%. Combining ultrasonography with CA-125 improved diagnostic accuracy to 91.32% (sensitivity 78.57%, specificity 93.75%), advancing preoperative triage by providing a simple, high-yield strategy for resource-limited settings.

## Background:

Ovarian tumours represent a wide range of neoplastic conditions due to apoplastic epithelial, stromal and germ-cell components, with each having its own biological, behavioural and prognostic characteristics [1]. Ovarian cancer is a major health concern in the world and it is the second most common female genital tract malignancy [2]. Its high mortality rate is greatly explained by the delayed diagnosis because most patients remain asymptomatic or have an unspecified abdominal symptom until the disease is advanced, forming in its advanced period. Ovarian cancer is the fifth most frequent cause of cancer mortality in women, with an annual global incidence of 239,000, resulting in approximately 152,000 deaths. The lifetime risk of developing ovarian cancer is 1 in 75 and the lifetime risk of dying is estimated at 1 in 100 [2]. The stage of the disease upon diagnosis has a great impact on prognosis. The five-year survival rates are high in early-stage disease (stage I), with more than 80% survival, but decrease significantly to less than 30% when women have advanced disease (stages III-IV) [3]. The clinical difficulty is presented by a deep location of the tumour in the pelvis, the lack of early warning signs and the similarity of gastrointestinal or gynecologic symptoms, including bloating, abdominal pain and early satiety [4]. As a result, preoperative diagnosis is usually incidental or delayed and it is necessary to have effective and convenient tools for preoperative diagnosis. The first-line imaging modalities used to examine adnexal masses include transabdominal and transvaginal ultrasonography [5].

Facets of morphology, such as mixed echogenicity, septations, solid components, papillary projections and ascites, which can help differentiate between malignant and benign pathology, can be evaluated using ultrasound. Despite the fact that ultrasonography is noninvasive, it is widely available and inexpensive; its diagnostic value can be affected by the experience of the operator and misinterpretations [4, 6]. The most used tumour marker in the assessment of ovarian cancer is serum CA-125, a high molecular weight glycoprotein that is a byproduct of the coelomic epithelium. High levels of CA-125 have been widely linked with epithelial ovarian malignancies and can assist in the risk stratification of malignancies. Although useful, CA-125 lacks cancer specificity and can be released in benign tumours, including endometriosis, pelvic inflammatory disease and benign cysts, which lead to low specificity [7]. Nonetheless, studies have shown that when CA-125 is used together with ultrasound results, the accuracy of diagnosis is significantly increased, particularly in postmenopausal women who present with suspicious lesions [8]. The integrative approach not only maximises the diagnosis process but also can inform the process of clinical decision-making and surgical planning. Because of the constraints of individual diagnostic methods and the practical use of tools in the low-resource environment, sonographic evaluation in conjunction with serum CA-125 could be the best way to maximise preoperative prediction of benign and malignant ovarian tumours. Therefore, it is of interest to show the diagnostic efficacy of ultrasonography and CA-125 individually and in combination with each other in distinguishing ovarian tumors by using histopathology as the reference standard.

## Materials and Methods:

This analytical cross-sectional study was conducted in the Department of Gynaecology and Obstetrics at Rangpur Medical College and Hospital, Bangladesh. The study was carried out over 12 months from January 2017 to December 2017. A total of 62 women clinically diagnosed with ovarian tumours were included. Following surgical intervention and histopathological assessment, participants were categorised into two groups: Group I (benign ovarian tumours, n=48) and Group II (malignant ovarian tumours, n=14).

## Inclusion criteria:

[1] Patients with suspected ovarian tumor diagnosed by history and clinical examination.

[2] Patient diagnosed with an ovarian tumour based on imaging (USG & /or CT scan).

## Exclusion criteria:

[1] Previously diagnosed and treated ovarian tumour (recurrent cases and patients on chemotherapy).

[2] Patients did not have an ultrasonography or serological testing of CA 125 preoperatively.

[3] Patients without histopathological examination.

## Data collection and study procedure:

Ethical approval was obtained from the institutional review committee and permission was granted by the Department of Gynaecology and Obstetrics. After providing detailed information about the objectives, risks and benefits, written informed consent was obtained from all participants. Data collection included structured interviews for demographic and clinical information, general and pelvic examinations and transabdominal ultrasonography assessing consistency, septation, papillary projections and ascites. Serum CA-125 estimation was performed using a cut-off value of 35 U/ml. Patients then underwent definitive surgical management and histopathological examination served as the diagnostic gold standard. Statistical analysis was conducted using SPSS version 23.0. Continuous variables were presented as means and standard deviations and compared using the Student t-test, while categorical variables were analysed using chi-square tests. Diagnostic indices, including sensitivity, specificity, accuracy, positive predictive value and negative predictive value, were calculated for ultrasonography, CA-125 and the combined approach. Confidentiality was properly maintained throughout the study.

## Results:

**Figure 1:**
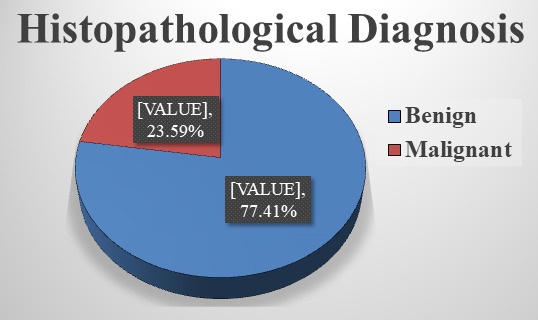
Proportion of benign and malignant cases, according to histopathology

Women with malignant ovarian tumours are older on average (49.14±16.59 years) than those with benign tumours (35.85±11.20 years) and this age difference is statistically significant (t=3.48, p=0.001). Use of oral contraceptive pills, ovulation-inducing drugs and family history did not differ significantly between benign and malignant groups (all p>0.05), suggesting these risk factors were not discriminative in this sample. Table 1 presents the demographic and baseline clinical characteristics of women diagnosed with benign and malignant ovarian tumours, including age distribution and key reproductive risk factors. Out of 62 ovarian tumour cases, 48 (77.41%) are benign and 14 (23.59%) are malignant, indicating that benign tumours constitute the majority in this hospital-based series. Figure 1 illustrates the overall distribution of benign and malignant ovarian tumours confirmed by histopathology in the study population. A total of 62 cases are included in this study. Among them 48 (77.41%) are benign and 14 (22.59%) are malignant. Ultrasonography correctly identifies malignancy with a sensitivity of 57.42% and a specificity of 89.50%, showing it is much better at ruling out malignancy than detecting all malignant cases. The overall diagnostic accuracy of USG is 82.17%, with a positive predictive value (PPV) of 61.84% and a negative predictive value (NPV) of 87.42%, indicating that a negative USG finding is relatively reliable, but positive findings still carry a notable false positive rate.

Table 2 shows the validity of ultrasonography in identifying benign and malignant ovarian tumours, based on true-positive, false-positive, false-negative and true-negative results. Serum CA-125 (cut-off 35 U/ml) shows higher sensitivity (71.42%) and specificity (93.75%) than USG alone, indicating better ability to detect malignancy and to correctly identify benign cases. CA-125 achieves an accuracy of 88.70%, with a PPV 76.92% and an NPV 91.83%, suggesting it is a stronger single test than USG for differentiating benign from malignant ovarian tumours in this cohort. Table 3 summarises the diagnostic performance of serum CA-125 levels in detecting ovarian malignancy using a cut-off value of 35 U/ml. When USG findings are combined with CA 125, sensitivity increases further to 78.57% and specificity remains high at 93.75%, improving detection of malignant cases while still accurately classifying benign lesions. The combined approach achieves the highest diagnostic accuracy of 91.32%, with both PPV and NPV at 78.57% and 93.75%, respectively; demonstrating that integrating imaging with a tumour marker provides the most reliable diagnostic performance among the evaluated strategies. Table 4 demonstrates the improved diagnostic accuracy achieved when ultrasonography findings are interpreted together with serum CA-125 levels.

## Discussion:

The present study evaluated the diagnostic performance of ultrasonography and serum CA-125, individually and in combination, in differentiating benign from malignant ovarian tumours, using histopathology as the reference standard. The findings highlighted important diagnostic patterns that align with and in several aspects of previously published research. The mean age of the malignant ovarian tumours was considerably more than that of the women who had benign tumours. This age distribution is consistent with findings reported by Mahale and others, who also noted a higher mean age in women with malignant tumours of the ovaries, indicating that age progression is one of the most important epidemiologic factors in the risk of ovarian cancer [5]. These same trends were observed in the study of Pegu *et al.* especially in postmenopausal women who had adnexal mass surgery [9]. These observations support the overall epidemiological assumption that the rate of increasing prevalence of ovarian cancer with age is steadily increasing, probably due to increased genomic instability and an increasing number of years of exposure to environmental risk factors [10]. Even though there is no significant difference in the use of oral contraceptive pills in the current research, prior pooled analyses have revealed protective associations. This trend is condoned by large-scale analyses of biomarker algorithms, which educated contraceptive use into the risk stratification systems, owing to its long-term protection [11]. The non-significant difference in the current data could be due to a small sample size, but not to the biological insignificance of the effect. In terms of clinical presentation, the malignant cases exhibited more abdominal distension and major weight loss. The findings are in line with case-based and clinical cohort reports of malignant ovarian tumours as more probable to manifest systemically through ascites, tumour burden, or endocrine changes [12, 13]. Abdominal pain was present in both benign and malignant conditions, meaning that it is nonspecific, which is also found in the literature, which states the overlapping appearances of symptoms in adnexal masses [14]. Ultrasonographic morphology was important in the differentiation of tumour type. Malignant tumours more often had complicated aspects such as mixed cystic-solid areas, septations, papillary projections and ascites. These characteristics are very much consistent with the high-risk sonography findings described in the IOTA Simple Rules and O-RADS systems, both of which are regularly characterised by the occurrence of irregular solid structures and multi-locular masses as predictors of malignancy [7]. In the study, the sensitivity of ultrasonography was 57.42 and the specificity was 89.50. Although the specificity is concordant with that of colour Doppler-enhanced sonography, the sensitivity was lower and this could be attributed to the use of transabdominal instead of transvaginal imaging, as mentioned by Mahale *et al.* who found higher sensitivity figures using enhanced Doppler evaluation [5, 10]. The variation can be due to the quality of a machine, experience and transabdominal over transvaginal ultrasound, which is normally more resolute. The mean difference in serum CA-125 levels was significant between the malignant group and normal cases and the elevation of serum CA-125 levels was significant in malignant cases. This diagnostic use of CA-125 reported in the current results is in line with previous large cross-sectional and case-series studies, as they are uniformly reporting moderate sensitivity but high specificity of the present marker in epithelial ovarian malignancy [6, 15].

Limitations associated with CA-125 were also apparent, as there are some cases of benign occurrences that demonstrated high levels. False-positive increases such as these have been widely reported in noncancerous gynaecological conditions, such as functional cysts, benign lesbians where leiomyomas, endometriosis and hormonally active lesions of the ovaries [12, 16]. A combination of the ultrasonographic morphology and CA-125 to achieve the best diagnostic performance, the sensitivity improved to 78.57 percent and the specificity was 93.75 per cent. This improved accuracy is consistent with recent multimodal diagnostic studies, reports of studies that combine CA-125 with new biomarkers or algorithmic models like ROMA and CPH-I [3, 17]. Similarly, Agrawal & Gunjan reported that when ultrasound findings suggestive of malignancy combined with elevated CA-125 levels, the specificity increased to 88%, with a positive predictive value of 92% and a negative predictive value of 85%. This combined approach significantly improved diagnostic accuracy, reducing the number of unnecessary surgeries for benign conditions [18]. Even though more recent biomarkers, including HE4 and TK1 protein, might be more effective on their own, several comparative studies suggest that CA-125 still has much potential when used in combination with ultrasound, especially when resources to more sophisticated assays are still limited [11, 17]. The combination methodology overcomes the shortcomings of each modality and the ultrasound is added, providing spatial and morphological evaluations and CA-125 is used to corroborate the overall diagnostic finding [19]. The ROC analysis in this study also supported the usefulness of CA-125, with an AUC of 0.931, which is statistically excellent in terms of discriminative ability. Similar AUCs have been achieved in algorithm tests of ovarian cancer detection based on biomarkers, which supports the clinical significance of CA-125 despite the advent of novel markers [3, 19]. The histopathological distribution in the present study, where serous cystadenoma is the most frequent benign tumour and serous cystadenocarcinoma is the most widespread malignant form, fits the conventional patterns in ovarian pathology. The dominance of epithelial tumours is similarly reported in experimental and clinical studies, which supports the validity of the current results in the extended spectrum of epidemiology [20, 21]. The study overall supports the clinical utility of both CA-125 and ultrasonography in preumber of ovarian tumours, although it suggests a much better diagnostic accuracy when the two are used together. Despites the fact that ultrasonography is an important method because it is accessible and specific CA-125 has an important biochemical dimension. The results confirm the previous evidence and highlight the necessity of considering both morphological and biochemical measurements in daily practice, especially in settings where sophisticated imaging technologies cannot be regularly used. There were some limitations in the study. The study was conducted in a single centre with a small sample size, which may limit the generalizability of the findings. Only transabdominal ultrasonography was used; more advanced methods such as transvaginal or Doppler studies were not available. Other tumour markers besides CA-125 could not be assessed due to limited laboratory resources, restricting broader diagnostic comparison.

## Conclusion:

There was high specificity with ultrasonography and increased sensitivity with CA-125, with diagnostic accuracy being improved significantly with a combination of both. Both modalities assessed histopathology, which supported their importance in preoperative assessment. The integrated method provides a combination of a viable and convenient technique for early detection of malignant ovarian tumours in the resource-limited environment.

## Figures and Tables

**Table 1:** Baseline characteristics of the study population

**Risk factors**	**Group I (N=48)**	**Group II (N=14)**	**t / χ^2^**	**P-value**
Age (Mean ± SD)	35.85 ± 11.20	49.14±16.59	3.48	0.001
Oral pill users	22(45.8%	6(42.8%)	0.03	>0.05
Ovulation-inducing drugs	5(10.40%)	1(7.14%)	0.13	>0.05
Family history	1(2.08%)	1(7.14%)	0.89	>0.05
Group I= Benign ovarian tumour;
Group II= Malignant ovarian tumour

**Table 2:** Correlation between histopathological diagnosis and ultrasonography findings

**USG features**	**Histopathological diagnosis**	
	**Malignant (n= 14)**	**Benign (n=48)**
Positive (n= 13)	8	5
	(True Positive)	(False positive)
Negative (n= 49)	6	43
	(False negative)	(True negative)
Diagnostic Performance of USG		
Sensitivity		57.42%
Specificity		89.50%
Positive predictive value		61.84%
Negative predictive value		87.42%
Accuracy		82.17%

**Table 3:** Correlation between histopathological diagnosis and serum CA-125 Levels

**CA-125 level**	**Histopathological diagnosis**	
	**Malignant (n= 14)**	**Benign (n=48)**
Positive (n= 13)	10 (True Positive)	3 (False positive)
Negative (n= 49)	4 (False negative)	45 (True negative)
**Diagnostic Performance of CA-125**		
Sensitivity		71.42%
Specificity		93.75%
Positive predictive value		76.92%
Negative predictive value		91.83%
Accuracy		88.70%

**Table 4:** Diagnostic performance of combined ultrasonography and CA-125

**USG features+CA125**	**Histopathological diagnosis**	
	**Malignant (n= 14)**	**Benign (n=48)**
Positive (n= 14)	11 (True Positive)	3 (False positive)
Negative (n= 48)	3 (False negative)	45 (True negative)
**Diagnostic Performance of Combined Testing**		
Sensitivity		78.57%
Specificity		93.75%
Positive predictive value		78.57%
Negative predictive value		93.75%
Accuracy		91.32%
